# Glial Purinergic Signals and Psychiatric Disorders

**DOI:** 10.3389/fncel.2021.822614

**Published:** 2022-01-05

**Authors:** Schuichi Koizumi

**Affiliations:** ^1^Department of Neuropharmacology, Interdisciplinary Graduate School of Medicine, University of Yamanashi, Yamanashi, Japan; ^2^GLIA Center, University of Yamanashi, Yamanashi, Japan

**Keywords:** astrocytes, ATP, microglia, depression, gliotransmitter

## Abstract

Emotion-related neural networks are regulated in part by the activity of glial cells, and glial dysfunction can be directly related to emotional diseases such as depression. Here, we discuss three different therapeutic strategies involving astrocytes that are effective for treating depression. First, the antidepressant, fluoxetine, acts on astrocytes and increases exocytosis of ATP. This has therapeutic effects via brain-derived neurotrophic factor-dependent mechanisms. Second, electroconvulsive therapy is a well-known treatment for drug-resistant depression. Electroconvulsive therapy releases ATP from astrocytes to induce leukemia inhibitory factors and fibroblast growth factor 2, which leads to antidepressive actions. Finally, sleep deprivation therapy is well-known to cause antidepressive effects. Sleep deprivation also increases release of ATP, whose metabolite, adenosine, has antidepressive effects. These independent treatments share the same mechanism, i.e., ATP release from astrocytes, indicating an essential role of glial purinergic signals in the pathogenesis of depression.

## Introduction

The human brain is estimated to contain ~100 billion neurons, which are connected by more than 10^14^ synapses to form complex neuronal networks. However, in addition to neurons, there are 5–10 billions of endotheliocytes and 80–85 billions of glial cells in the human brain (von Bartheld et al., 2016; Butt and Verkhratsky, 2018), and they come into contact with synapses, axons and dendrites, to communicate with neurons and regulate the function and structure of neuronal networks. Therefore, it is becoming clear that transmission and processing of information, which is the core function of the brain, is carried out by not only neuronal activity but also by communication between neurons and glial cells (Haydon, 2001). In particular, astrocytes have a large number of fine processes consisting of branches and leaflets (Semyanov and Verkhratsky, 2021), and one astrocyte can contact hundreds of thousands of synapses to maintain extracellular ion homeostasis, remove excess neurotransmitters, release gliotransmitters, and supply energy. Astrocytes can, therefore, actively control the activity of neurons (Eroglu and Barres, 2010; Khakh and Sofroniew, 2015). Initially, it was reported that astrocytes form tripartite synapses with neurons to control synaptic transmission, but the latest concept is “active milieu,” which is based on the dynamic interposition and interaction among compartments of neurons, astrocytes, oligodendrocytes, microglia, blood vessels, extracellular space and extracellular matrix. The active milieu would greatly affect psychiatric diseases (Semyanov and Verkhratsky, 2021). In addition to such dynamic control of synaptic transmission, it is clear that astrocytes can modulate neural networks by controlling synapse formation and elimination (Kim et al., 2011, 2016). Therefore, glial cell abnormalities may be associated with various psychiatric disorders and neurodegenerative disorders. Here, we review abnormalities in communication between astrocytes and neurons via extracellular ATP (ATPo) and discuss this within the context of the molecular pathology of depression. We also discuss how antidepressants or other treatments exert therapeutic effects on astrocytes.

Glial cells can be broadly classified into three types, i.e., astrocytes, oligodendrocytes, and microglia. Astrocytes the largest cells and occupy 20–40% of all glial cells, which depends on the brain regions (Verkhratsky and Nedergaard, 2018), and play a role in supporting neuronal functions, such as physical support of neurons, removal of waste products and excess neurotransmitters, and the supply of nutrition from blood vessels to neurons. Oligodendrocytes form myelin sheaths and enable jumping conduction, and microglia behave as immunocompetent cells in the brain. Although it is clear that glial cells play essential roles in the brain, the word “glia,” originated from Greek, was named by Virchow (1958), showing connective tissues, which are electropysiologically non-excitable and were thought to be cells that simply fill in between the neurons and blood vessels, they have been far less studied than neurons. However, with the recent rapid development of neuroscience, this understanding has dramatically changed.

As mentioned above, glial cells are electrically non-excitable cells, but other biochemical indicators, such as Ca^2+^ signals, show that glial cells, especially astrocytes, are highly excitable (Ca^2+^ excitability). In addition, glial cells express various neurotransmitter receptors, ion channels, and transporters, and have the ability to transmit information by releasing chemicals such as ATP (Koizumi et al., 2003), which are linked to Ca^2+^ excitability. In other words, glial cells are capable of receiving, processing, and transmitting information, i.e., gliotransmission (Haydon, 2001). Glial cells actively communicate with neurons in a bidirectional manner, regulate neuronal functions, and propagate information in a manner that differs from neurons in time and space. The role of glial cells in brain function has become the focus of much attention because of the revelation that glial cells transmit and process information.

## Astrocytes, Extracellular ATP and Depression

Depression has a wide range of symptoms and involves complex dysfunction of many neuronal systems, particularly involving monoaminergic neurons (Schildkraut, 1967; Nestler et al., 2002). Brain imaging studies have revealed that certain brain regions, such as the hippocampus and prefrontal cortex, have decreased brain volume in depressed patients (Bremner et al., 2002; Sheline, 2003). These volume changes have been suggested to result from changes in glial cells, such as astrocytes, rather than from functional changes in neurons (Banasr and Duman, 2008; Murphy-Royal et al., 2019). In addition, many studies using postmortem brains of depressed patients have shown that the number of glia is reduced in the cerebral cortex (Miguel-Hidalgo et al., 2000; Cotter et al., 2001). Despite the fact that antidepressants and other therapies act in part on astrocytes and other glial cells in the brain, little is known about their mechanisms of action and the causal relationship between glial cell responses and antidepressive effects.

Diffusible molecules such as neurotransmitters, gliotransmitters, cytokines etc. in the brains of mice that readily exhibit depressive behaviors under stress were compared with those from mice that are resistant to stress. The most differentially abundant molecule was ATPo and the decreased level of ATPo in the depression-exhibiting stressed mice was not just a result of stress loading, but also a cause of depression induction (Cao et al., 2013). In addition, astrocytes were shown to be an important source of ATPo. Insulin enhances ATP release by stimulating insulin receptors (IRs) in astrocytes. This function is impaired in diabetic patients, which may account for their increased risk of depression. Indeed, IR-deficient animals exhibit depressive-like and anxiety-like behaviors (Cai et al., 2018). In both cases, depressive-like behaviors disappear when ATP is administered to increase either extracellular ATP or its metabolite adenosine in the brain (Cao et al., 2013; Cai et al., 2018). Therefore, a decrease in ATPo is a cause of depressive-like behavior. As mentioned above, ATPo plays a central role as a gliotransmitter released from astrocytes and dynamically regulates neuronal and glial cell functions (Koizumi et al., 2003). Therefore, these findings strongly indicate that the molecular pathogenesis of depression is caused in part by the decreased function of astrocytes (ATP release capacity), and that a target of antidepressant drugs is astrocytes. Therefore, improvement of their function may exert therapeutic effects.

## The Antidepressant Fluoxetine and Astrocytes

We have shown that the selective serotonin reuptake inhibitor (SSRI), fluoxetine (FLX), increases ATPo from primary cultured astrocytes in a concentration-dependent manner (Kinoshita et al., 2018). When treated with FLX both *in vivo* and *in vitro*, FLX causes ATP release from astrocytes through VNUT (vesicular nucleotide transporter)-dependent exocytosis. However, this effect of FLX was not observed in VNUT-deficient (VNUT-KO) mice. Therefore, it is clear that FLX exocytoses ATP via VNUT. To clarify whether this FLX-induced increase in ATPo is a necessary condition for antidepressant effects, we measured the immobility time in the tail suspension test. Chronic administration of FLX caused a strong antidepressant effect (shortening of immobility time) in wild-type mice, but no such effect was observed in VNUT-KO mice. The antidepressant effect of FLX was also abolished in astrocyte-specific VNUT-deficient mice. Furthermore, mice with astrocyte-specific overexpression of VNUT showed antidepressant effects with lower concentrations of FLX. These results strongly indicate that chronic administration of FLX enhances VNUT-dependent ATP release from astrocytes, and that elevation of ATPo is a necessary condition for the antidepressant effect of FLX.

Chronic administration of antidepressants increases the expression of various trophic factors, such as GDNF and FGF (Takebayashi et al., 2006; Kajitani et al., 2012). In particular, increased expression of brain-derived neurotrophic factor (BDNF) has attracted much attention as a mechanism for the response to antidepressants (Nibuya et al., 1995). BDNF is expressed predominantly in neurons, moderately in microglia, but rarely in astrocytes. In the hippocampus and cerebral cortex, BDNF is expressed almost exclusively in neurons (Schmidt-Kastner et al., 1996; Gorba and Wahle, 1999). Chronic FLX treatment for three weeks greatly enhances BDNF expression in hippocampal pyramidal cell layer and dentate gyrus neurons. This antidepressant-induced increase in BDNF expression was also observed in astrocytes. In primary cultured hippocampal astrocytes, FLX increases *Bdnf* gene expression and BDNF protein levels. Although the serotonin transporter (Hirst et al., 1998) and the noradrenaline transporter (Inazu et al., 2003) are expressed in astrocytes, the enhancement of BDNF expression by FLX was independent of their suppression. However, BDNF expression was suppressed by antagonists of the ATP P2Y11 receptor and the adenosine A2b receptor. Thus, ATP released from astrocytes by FLX acts on P2Y11 receptors as an autocrine signal and on A2b receptors as adenosine, which is rapidly degraded by extracellular ATPase, to stimulate BDNF expression.

Both P2Y11 and A2b receptors are Gs-coupled receptors, and their stimulation enhances intracellular cAMP signaling. In addition, protein kinase A is involved in the phosphorylation of CREB and increased the expression of VNUT. Therefore, ATP autocrine signaling is a positive feedback signal that further enhances ATP release. Very interestingly, paroxetine, another SSRI-type antidepressant, and imipramine, a tricyclic antidepressant, also had the above-mentioned effects on BDNF production in astrocytes. Thus, the BDNF-producing effect of astrocytes may not be specific to FLX, but may be a common pharmacological effect of antidepressants.

**Figure 1:**
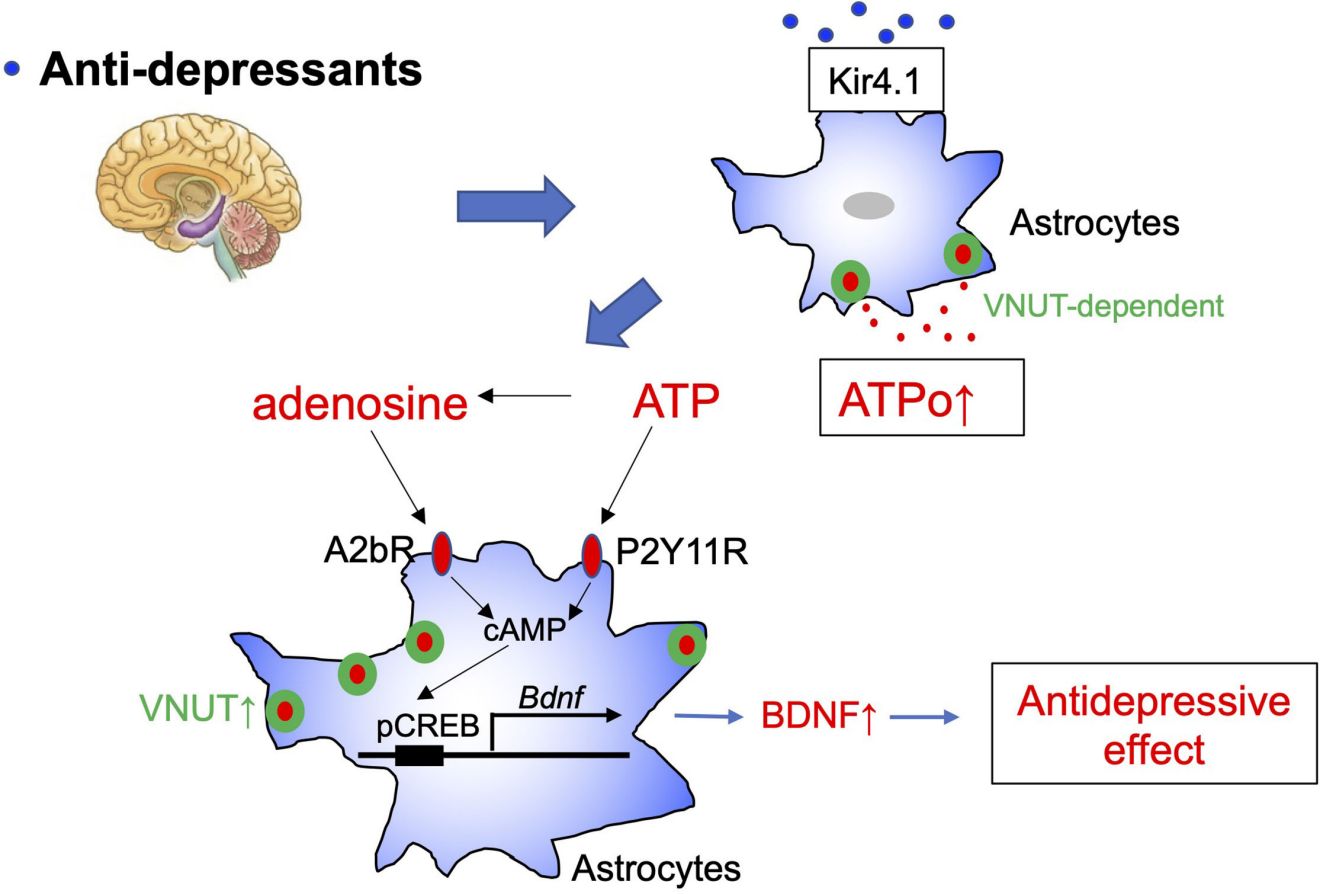
Schematic diagram of the antidepressant, fluoxetine, and astrocytes. Fluoxetine (FLX) increases vesicular nucleotide transporter (VNUT)-dependent ATP exocytosis from astrocytes and causes elevation of extracellular ATP (ATPo). Kir4.1 may be an important target molecule of FLX in astrocytes. Extracellular ATP and its metabolite, adenosine, act on P2Y11 and A1b receptors to increase cAMP levels and enhance *Bdnf* transcription in astrocytes via CREB phosphorylation. The BDNF produced and released by astrocytes is thought to cause synapse and network remodeling and to exhibit antidepressant effects. cAMP may also enhance VNUT expression via protein kinase A, which further strengthens ATP/adenosine signaling.

It was recently demonstrated that overexpression and overactivity of Kir4.1 in hippocampal habenula astrocytes is responsible for depressive-like behavior (Cui et al., 2018). Kir4.1 is an inwardly rectifying K^+^ channel mainly expressed in astrocytes and oligodendrocytes surrounding synapses and blood vessels in the cortex, thalamus, hippocampus, and brain stem. Kir4.1 primarily controls the resting membrane potential of astrocytes and maintains the extracellular ionic and osmotic environment by promoting K^+^ transport from regions of high extracellular K^+^, which results from synaptic excitation, to those of low extracellular K^+^. Very interestingly, many SSRI and tricyclic antidepressants, including FLX, inhibit Kir4.1 (Ohno et al., 2007). Kir4.1 is also a regulator of BDNF production in astrocytes (Ohno et al., 2018). Although further studies are needed, these results indicate that FLX may inhibit Kir4.1 in astrocytes, thereby enhancing ATPo, BDNF production, and antidepressant effects (Figure 1).

## Electroconvulsive Therapy and Astrocytes

**Figure 2:**
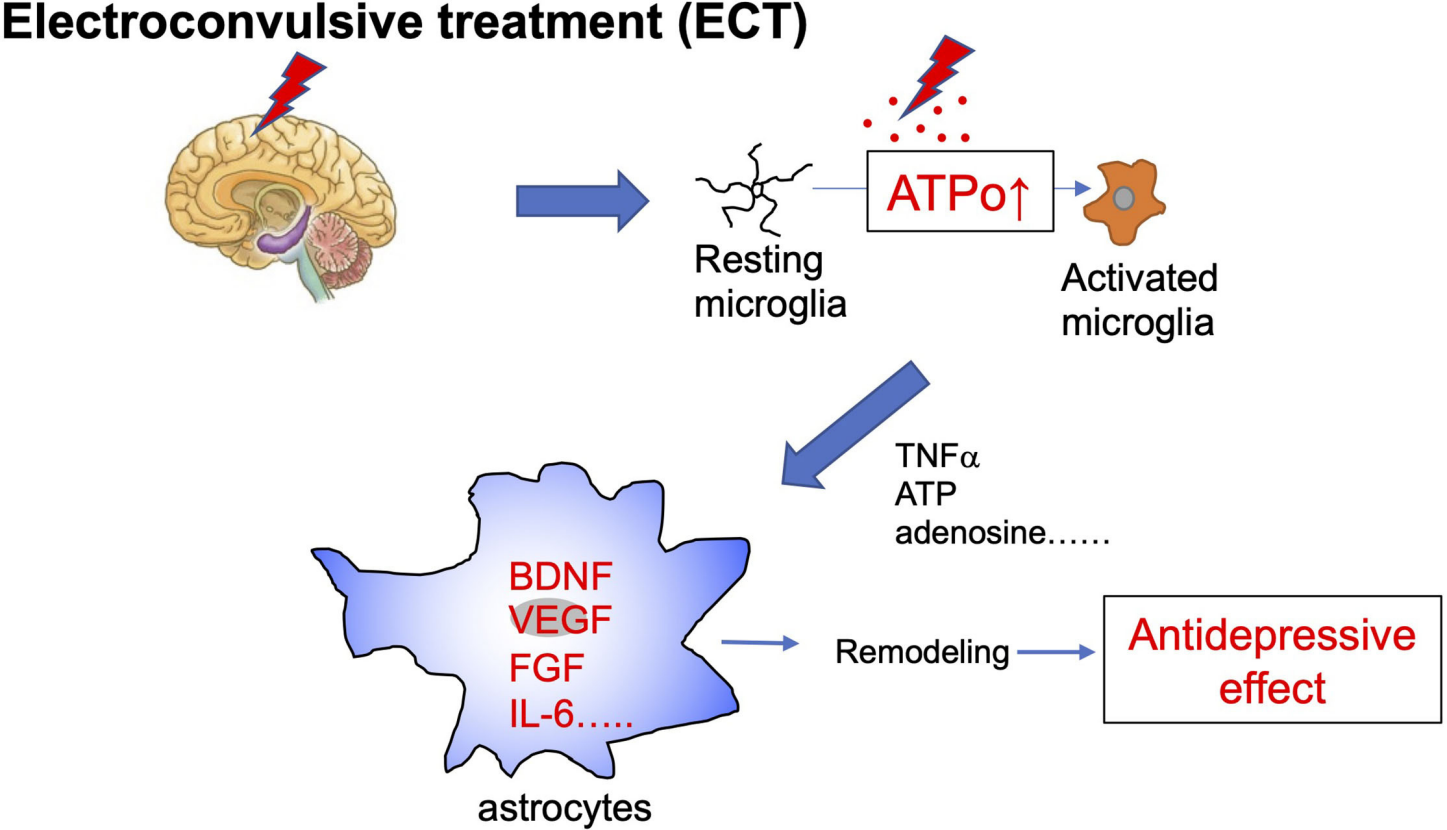
Schematic diagram of electroconvulsive therapy and astrocytes. Electroconvulsive therapy (ECT) increases extracellular ATP (ATPo), which in turn activates microglia from a resting to an activated phenotype. Acute ECT causes the transient activation of microglia and increases levels of many active molecules, such as proinflammatory cytokines and ATP. These diffusible molecules produced by activated microglia act on further astrocytes and cause their activation. Activated astrocytes increase levels of trophic factors, such as BDNF, VEGF, FGF, and also cytokines, such as IL-6, which cause synapse and network remodeling. These astrocyte events are involved in antidepressive effects.

Electroconvulsive therapy (ECT) is a treatment in which an electric current is applied to the scalp to cause convulsions. This therapy is highly effective for psychiatric disorders, especially drug-resistant disorders such as depression. ECT is a very effective and fast-acting treatment; therefore, its molecular mechanisms have been extensively studied. For example, the effects of ECT on neurotransmitters and their receptors, as well as on intracellular signal transduction systems, i.e., monoamines, cortisol, adrenocorticotropin-releasing hormone, corticotropin-releasing factor, thyroid-stimulating hormone, prolactin, oxytocin, and vasopressin have been studied. Recently, ECT was shown to increase BDNF levels (Rocha et al., 2016), indicating that ECT may have a neurotropic effect and increase the remodeling of brain networks. ECT also increases hippocampal neurogenesis (Madsen et al., 2000) and gliogenesis (Wennstrom et al., 2006), and elevates other trophic factors, such as nerve growth factor (Conti et al., 2009), and fibroblast growth factor (Elfving and Wegener, 2012). By these actions, ECT is suggested to remodel structural connections of neuronal networks and neuronal function, thereby causing its therapeutic effect. ECT also affects immune responses (Guloksuz et al., 2014; An and Shi, 2020) by modulating microglia and astrocytes and altering certain cytokines, such as tumor necrosis factor-α (Hestad et al., 2003) and interleukin-6 (IL-6) (Hestad et al., 2003), which are also associated with brain remodeling. For these glia-mediated immune responses, extracellular ATP, its metabolite adenosine, and their corresponding receptors have a pivotal role. Consistently, ATP is released in response to ECT or electrical convulsive stimulation (ECS) in animal models (van Calker and Biber, 2005; Sadek et al., 2011; Maruyama et al., 2020). The metabolism of ATP to adenosine is very rapid; therefore, an increase in ATPo may be an initial event that triggers ECT-evoked responses. Acute ECT/ECS rapidly activates microglia and their related proinflammatory cytokines, thereby leading to neuroinflammation. Such microglial activation is thought to be neuroprotective because it is transient and can protect neurons. Importantly, microglial activation is followed by activation of astrocytes (An and Shi, 2020). Activated astrocytes produce further growth factors, such as BDNF (Nibuya et al., 1995), and cytokines, such as IL-6. The antidepressive effect of ECT may result from activating this microglia-astrocyte communication and resetting abnormal functional or structural connections to normalize brain functions (Figure 2).

## Sleep Deprivation Therapy and Astrocytes

In addition to ECT, sleep deprivation (SD) therapy is a non-pharmacological therapy that is used to treat psychiatric diseases such as major depression. SD was first reported by (Pflug and Tolle, 1971a,b), and has been used mainly in Europe and the United States for the treatment of intractable depression. When depressed patients were kept awake overnight, it was found that their depressive symptoms improved significantly. It should be noted that while drug therapy takes more than two weeks to show an effect, the response to SD is usually rapid. SD is considered to be effective for drug-resistant and intractable depression and does not have any severe side effects, unlike antidepressant therapy. Therefore, SD has received much attention as a therapy for depression, but its mechanisms of action remain largely unknown.

**Figure 3:**
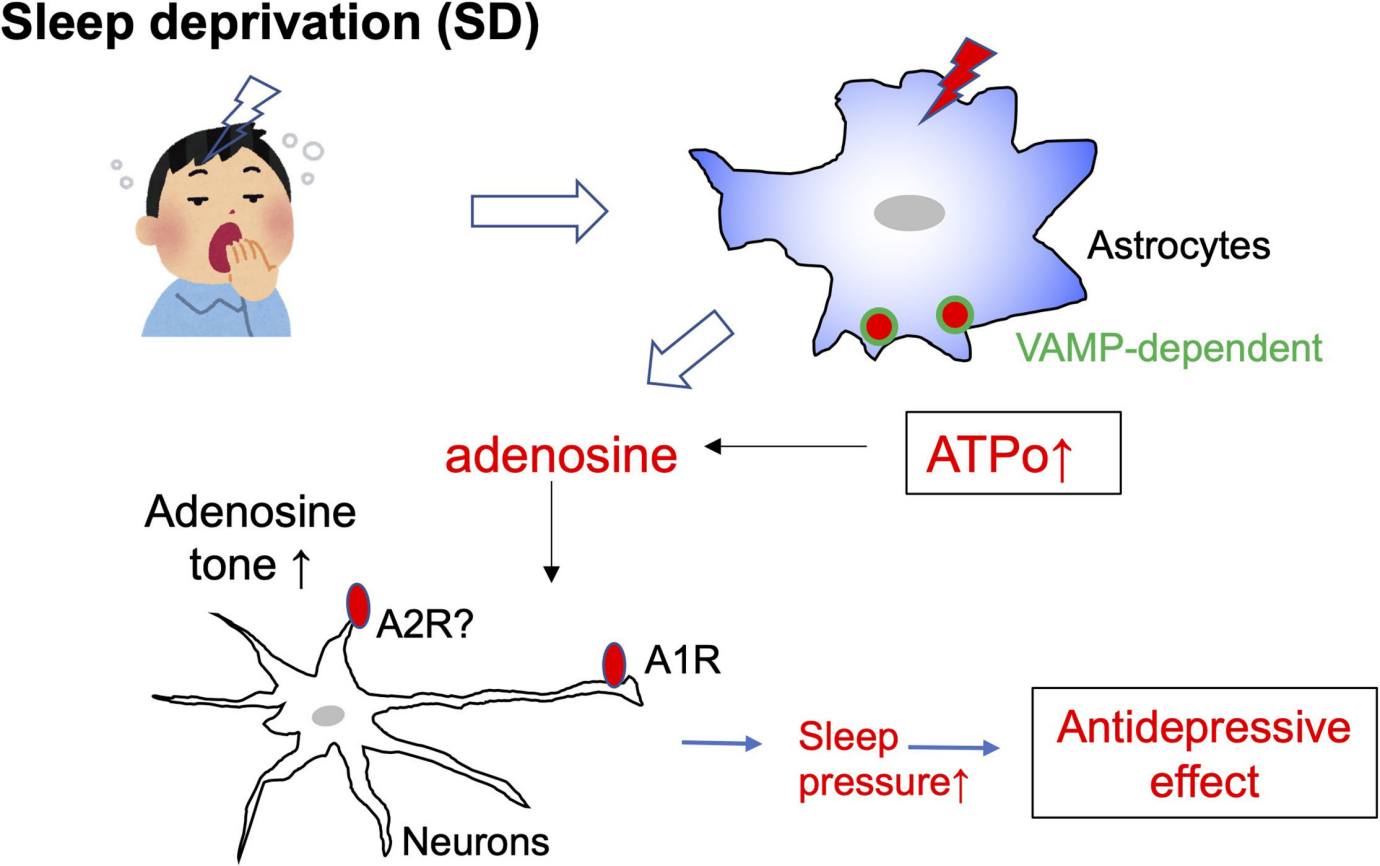
Schematic diagram of sleep deprivation and astrocytes. Sleep deprivation (SD) increases the exocytosis of ATP from astrocytes via vesicle-associated membrane protein (VAMP)-dependent mechanisms. The elevated extracellular ATP (ATPo) is quickly converted to adenosine, and the tone of adenosine is increased. Adenosine acts on A1 receptors in neurons and increases sleep pressure. An increase in sleep pressure by adenosine/A1 receptors is highly involved in the antidepressive effect.

SD was recently demonstrated to facilitate adenosine signaling (Hemmeter et al., 2010; Dallaspezia and Benedetti, 2015), i.e., SD increases vesicle-associated membrane protein (VAMP)-dependent ATP exocytosis from astrocytes (Hines et al., 2013). The effect of adenosine was mimicked by administration of an adenosine precursor, S-adenosyl-L-methionine (De Berardis et al., 2013). In addition, depressed patients show decreased levels of purines (Renshaw et al., 2001). These findings indicate that a decreased level of adenosine may be a cause of depression, and thus elevation of adenosine levels either endogenously or exogenously produces antidepressive effects. Adenosine is sleep inducing, and SD increases adenosine tone, which in turn increases sleep pressure. It is still unknown which molecules and mechanisms underlie the sleep pressure-induced antidepressive effect; however, a key molecule may be the adenosine A1 receptor. SD is associated with upregulation of adenosine A1 receptors which may provide a novel pathway for antidepressant development (Hines et al., 2013). The importance of A1 receptors was confirmed using transgenic mice that have enhanced doxycycline-upregulated A1 receptors. These mice were more resilient to depressive-like behavioral changes in a chronic depression model (Serchov et al., 2015). Adenosine is released from either neurons or glial cells, but it is thought that the majority of adenosine is produced by the degradation of ATPo released from astrocytes or other cells (Hines et al., 2013). Therefore, although the detailed mechanisms of the SD-induced antidepressive effect await clarification, astrocytes and ATP/adenosine-mediated signals are closely involved in its therapeutic effect (Figure 3).

## Conclusion

In this review, we explored the molecular pathogenesis of depression by assessing the common features of effective therapeutic drugs and methods for treating depression. In particular, we identified astrocyte phenomena commonly induced by the antidepressants FLX, ECT, and SD, and concluded that increased levels of ATPo is a common mechanism. ATPo increase is a very early event that is caused by release or leakage in response to various stimuli, which transmits information to the surrounding environment. Glial cells play a key role in the release and reception of ATP. However, almost all these findings, i.e., a decrease in ATP and purinergic system is associated with the molecular pathogenesis of depression (Cao et al., 2013), antidepressants (Kinoshita et al., 2018), ECT (Maruyama et al., 2020) and SD (Hines et al., 2013) have antidepressive effects by increasing ATP, adenosine, and purinergic system, were assessed by *in vivo* animal models. The relevance of ATP and the purinergic system to the pathophysiology of actual depressed patients remains unclear. Therefore, future studies using postmortem brain, human brain imaging, and human brain using PET will be necessary to clarify these issues. The pathophysiology of depression varies widely, and it is a complex disease in which various molecules and cells are involved. However, it is highly likely that ATP and astrocytes affect the complex cascades involved in the initial pathology of depression. Focus on these factors will further the development of drugs for depression that target glial cells.

## Author Contributions

The author confirms being the sole contributor of this work and has approved it for publication.

## Funding

This work was supported by JSPS Grants-in-Aid for Scientific Research (KAKENHI) (JP25117003, 18H0512, 19H04746, 20H05060, 20H05902, 21H04786, and 21K19309), AMED-CREST (JP21gm1310008), the Takeda Science Foundation, The Mitsubishi Foundation and the Frontier in Brain Science Program of Yamanashi University.

## Conflict of Interest

The author declares that the research was conducted in the absence of any commercial or financial relationships that could be construed as a potential conflict of interest.

## Publisher's Note

All claims expressed in this article are solely those of the authors and do not necessarily represent those of their affiliated organizations, or those of the publisher, the editors and the reviewers. Any product that may be evaluated in this article, or claim that may be made by its manufacturer, is not guaranteed or endorsed by the publisher.
